# Plakophilin3 Loss Leads to an Increase in PRL3 Levels Promoting K8 Dephosphorylation, Which Is Required for Transformation and Metastasis

**DOI:** 10.1371/journal.pone.0038561

**Published:** 2012-06-06

**Authors:** Nileema Khapare, Samrat T. Kundu, Lalit Sehgal, Mugdha Sawant, Rashmi Priya, Prajakta Gosavi, Neha Gupta, Hunain Alam, Madhura Karkhanis, Nishigandha Naik, Milind M. Vaidya, Sorab N. Dalal

**Affiliations:** 1 KS215, Advanced Centre for Treatment Research and Education in Cancer (ACTREC), Tata Memorial Centre, Kharghar Node, Navi Mumbai, Maharashtra, India; 2 Pharmacology Department, Piramal Life Sciences Ltd., Mumbai, Maharashtra, India; Northwestern University Feinberg School of Medicine, United States of America

## Abstract

The desmosome anchors keratin filaments in epithelial cells leading to the formation of a tissue wide IF network. Loss of the desmosomal plaque protein plakophilin3 (PKP3) in HCT116 cells, leads to an increase in neoplastic progression and metastasis, which was accompanied by an increase in K8 levels. The increase in levels was due to an increase in the protein levels of the Phosphatase of Regenerating Liver 3 (PRL3), which results in a decrease in phosphorylation on K8. The increase in PRL3 and K8 protein levels could be reversed by introduction of an shRNA resistant PKP3 cDNA. Inhibition of K8 expression in the PKP3 knockdown clone S10, led to a decrease in cell migration and lamellipodia formation. Further, the K8 PKP3 double knockdown clones showed a decrease in colony formation in soft agar and decreased tumorigenesis and metastasis in nude mice. These results suggest that a stabilisation of K8 filaments leading to an increase in migration and transformation may be one mechanism by which PKP3 loss leads to tumor progression and metastasis.

## Introduction

Changes in cytoskeletal architecture and cell-cell adhesion are often observed in cells undergoing neoplastic transformation. Desmosomes are adherens type junctions that are required for cell-cell adhesion, especially in tissues that experience mechanical stress and anchor intermediate filaments (IF’s) leading to the generation of a tissue wide IF network (reviewed in [1], [2], [3]). IF’s are an important component of the cytoskeleton that give shape and rigidity to cells and are comprised of the type I (acidic K9–K28) and type II (basic K1–K8 and K71–74) subtypes in epithelial cells [4],[5]. The keratins are expressed in pairs of type I and type II keratins in a tissue specific and differentiation dependent manner [6], [7], [8] e.g. simple epithelia express the keratin pair K8 and K18 [9],[10], while all stratified epithelia express K5 and K14 in the basal layer [10], [11].

The aberrant over-expression of K8 and K18 has been observed in a number of squamous cell carcinomas irrespective of their origin [9], [12], [13], [14]. Over-expression of these two proteins is also associated with increased invasive and migratory properties [15], [16] and with poor prognosis [17]. Thus, increased expression of K8/18 could lead to tumor formation. Over-expression of K8 in the immortal foetal buccal mucosal cell line, FBM, led to increased transformation in vitro and in vivo [18]. Conversely, a decrease in K8 and K18 levels leads to a decrease in transformation in tumor cell lines derived from stratified epithelia due to alterations in α6β4 integrin signalling [19]. A knockdown in K8 leads to decreases in α6β4 levels which are accompanied by a decrease in invasion, transformation and α6β4 mediated signalling.

Metastasis in colon cancer often correlates with an increased expression of the Phosphatase of Regenerating Liver -3 (PRL-3) [20], [21]. In addition, PRL-3 expression inhibits PTEN and PI3K mediated signalling and leads to the loss of proteins such as E-cadherin and γ-catenin, which are often associated with activation of the Epithelial Mesenchymal Transition (EMT) program [22]. While these data suggested that PRL3 expression could lead to metastasis, it was not clear what targets of PRL3 were required for metastatic progression. Mizzuchi et. al. demonstrated that PRL-3 expression led to dephosphorylation of K8 and this correlated with an increase in metastatic progression in colon tumors [23], suggesting that post-translational alterations on K8 could drive tumor progression. Further, data reported by Alam et. al. demonstrated that K8 dephosphorylation correlates with increased tumor progression in oral squamous cell carcinoma (OSCC) and could be used as a prognostic marker for OSCC progression [24].

Plakophilin3 (PKP3) is a desmosomal plaque protein that belongs to the p120 catenin sub family of the armadillo family of proteins and is found in desmosomes in most epithelial tissues with the exception of hepatocytes [25], [26]. PKP3 interacts with multiple desmosmal proteins as well as K18 [27] and is required for the recruitment of various desmosomal proteins to the cell border and the initiation of desmosome formation [28]. It has been suggested that loss of desmosome function leads to the acquisition of the neoplastic phenotype (reviewed in [29]). Consistent with the data that PKP3 is required for desmosome formation [28], PKP3 loss was associated with tumor progression and metastasis in tumors derived from the oral cavity and the colon [30], [31], [32]. A previous report from our laboratory has demonstrated that a decrease in the levels of PKP3 in HCT116 cells, which are derived from the colon [33], led to an increase in colony formation in soft agar and increased tumor formation and metastasis in nude mice. PKP3 has been shown to physically associate with keratins, specifically K18 [27], which is the obligate partner for K8 [4], [5]. The results in this report demonstrate that upon PKP3 loss, an increase in PRL3 levels is observed, which leads to increased dephosphorylation of K8 and an increase in K8 levels. Our results also suggest that the increase in K8 levels is required, at least in part, to mediate the tumor progression and metastasis induced upon PKP3 loss.

## Results

**Figure 1:**
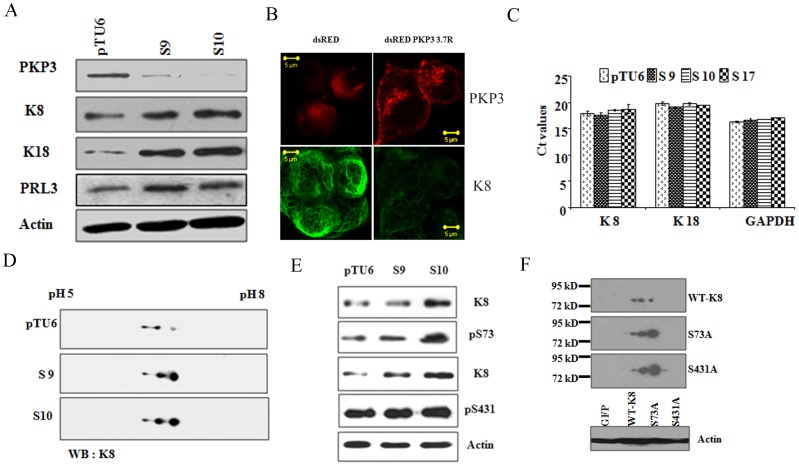
High K8 levels in the PKP3 knockdown clones are due to a decrease in phosphorylation. **A.** Protein extracts from the vector control (pTU6) and PKP3 knockdown clones (S9 and S10) were resolved on SDSPAGE gels and Western blots performed with the indicated antibodies. **B.** S9 cells were transfected with either dsRed or the shRNA resistant dsRed PKP3 3.7R cDNA. 48 hours post transfection cells were stained with antibodies to K8 (green) and visualized by confocal microscopy. Note that dsRed PKP3 3.7R localizes to the border as previously described (indicated by arrow) [28]. Original magnification is 630X with a 2X optical zoom. Scale bar 5 µm. **C.** A real time PCR analysis to determine the mRNA levels of K8 and K18 was performed on RNA isolated from the vector control and PKP3 knockdown clones. GAPDH was used as an internal control for normalization. The Ct values for all samples are shown on the Y-axis. **D.** Protein extracts from the PKP3 knockdown clones or the vector control were subjected to 2-dimensional gel electrophoresis and Western blots performed with antibodies to K8. **E.** Protein extracts from the vector control cells or the PKP3 knockdown clones were resolved on SDS-PAGE gels followed by Western blotting with antibodies to K8 or phosphospecific antibodies against S73 (α-S73), S431 (α-S431) and actin. **F.** HCT116 cells transfected with GFPK8 or GFPS73A or GFP S431A were resolved on two dimensional gels followed by Western blots with antibodies to K8. MW markers are indicated. Protein extracts from the transfected cells were resolved on SDS-PAGE gels followed by Western blotting with antibodies to actin to serve as loading controls.

Previous work from this laboratory has demonstrated that PKP3 loss in HCT116 cells leads to increased neoplastic progression and metastasis [33]. PKP3 has been shown to form a complex with K18 in a yeast two hybrid assay [27]. To determine if PKP3 forms a complex with K18 in HCT116 cells, protein extracts from HCT116 cells were incubated with either non-specific IgG or antibodies to K18. The immunoprecipitation reactions were resolved on SDS-PAGE gels followed by Western blotting with antibodies to K18 and PKP3. As shown in Figure S1A, complexes immunoprecipitated by the K18 antibody contain K18 and PKP3, in contrast to extracts incubated with the control antibody. As K8 and K18 are a keratin pair expressed mostly in simple epithelia [9], [10], the levels of K8 and K18 were determined in the HCT116 derived PKP3 knockdown clones S9 and S10 [33]. S9 and S10 were generated using two different shRNA constructs, both show similar defects in desmosome formation, and both show increased transformation and metastasis when injected into nude mice [28], [33]. Hence, all future experiments were performed with these two clones. A Western blot analysis demonstrated that the levels of both K8 and K18 were elevated in the PKP3 knockdown clones (S9 and S10) as compared to the vector control cells (pTU6) (figure 1A). Western blots for actin served as loading controls and a Western blot for PKP3 shows the decrease in PKP3 levels in S9 and S10. A high salt extraction shown in Figure S1B demonstrated that in addition to K8 and K18 one additional band was present that was recognized by an antibody that recognises most type I keratins (data not shown). The PKP3 knockdown clones, S9 and S10, showed an elevation of all three keratins (Figure S1B). The increased keratin levels did not affect filament formation by K8 or K18 (Figure S1C). The other band was not another intermediate filament protein like vimentin as a Western blot demonstrated that either vector control or knockdown cells derived from HCT116 cells do not express vimentin (Figure S1D) as previously reported [34], [35].

The two clones, S9 and S10, are generated using two different shRNA’s suggesting that the phenotypes observed are probably not due to off-target effects of the shRNA [33]. To determine whether the effects observed were due to PKP3 knockdown, the S9 cells were transfected with either dsRed alone or a PKP3 cDNA construct resistant to the 3.7 shRNA used to generate the S9 clone, dsRed PKP3 3.7R [28]. Post transfection the cells were stained with antibody to K8 and imaged by confocal microscopy. As shown in figure 1B, introduction of the shRNA resistant construct, dsRed PKP3 3.7R resulted in a decrease in keratin staining in comparison to cells transfected with the vector control. This is in agreement with previous work from our laboratories, which demonstrated that the introduction of an shRNA resistant PKP3 cDNA into these cell clones does reverse many of the phenotypes observed upon PKP3 knockdown [28].

To determine if an increase in RNA levels led to the increase in K8 protein in the PKP3 knockdown clones, a reverse transcriptase coupled Real Time PCR analysis was performed. The mRNA levels of K8 and K18 in the PKP3 knockdown clones were not significantly altered as compared to the vector control (pTU6) (figure 1C), therefore the increase was not due to an increase in mRNA levels. GAPDH levels were also measured in this assay as loading controls. To determine if the increase in protein stability was due to post-translational modifications on K8/K18, protein extracts from the vector control and PKP3 knockdown clones were subjected to two-dimensional gel electrophoresis. In these experiments, K8 migrated as three distinct spots in the vector control cells, with the most acidic spots being the most abundant (figure 1D, top panel). In contrast, in the PKP3 knockdown clones, the most basic spots increased in abundance with an overall increase in protein levels (figure 1D). The pattern observed is similar to the pattern observed in cells that over-express the protein phosphatase PRL3 [23], suggesting that the altered pattern may be associated with a decrease in phosphorylation in K8. No change in migration was observed for K18 (Figure S2A) though an increase in protein levels was observed.

Two major sites of phosphorylation, S73 and S431, have been reported in K8 [36], [37]. Western blots with phosphospecific antibodies for S73 and S431 demonstrated that the levels of K8 phosphorylated on S73 increase in the PKP3 knockdown clones as compared to the vector control, which is consistent with the increase in K8 expression (figure 1E). In contrast, the levels of K8 phosphorylated on S431 remained constant in the PKP3 knockdown clones when compared to the vector control, suggesting that there is a decrease in phosphorylation on K8 at S431 in the PKP3 knockdown clones (figure 1E). Western blots for actin were performed as loading controls. These results were quantitated by densitometry and when the levels of these individual proteins were normalized to the levels of actin, it was observed that while the levels of K8 and K8 phosphorylated on S73 were elevated in the PKP3 knockdown clones, the levels of K8 phosphorylated on S431 stayed constant suggesting that S431 was dephosphorylated upon PKP3 knockdown (Figure S2B). The specificity of the phospho-specific antibodies was confirmed by their inability to recognize a phospho-site mutant in a Western blot analysis (Figure S2C and [24]). The levels of p38MAPK, which phosphorylates S73 in response to stress [37], were similar in both the HCT116 derived vector control and PKP3 knockdown clones (Figure S2D), which is consistent with the observation that S73 phosphorylation is not altered in the PKP3 knockdown clones. A Western blot for 14-3-3γ served as a loading control (Figure S2D).

To further demonstrate that the change in migration and stability of K8 was due to changes in phosphorylation in K8, two phospho-site mutants (S73A and S431A) [36], [37] and WT K8 were expressed as GFP fusions in HCT116 cells. Protein lysates were resolved on two dimensional gel electrophoresis followed by Western blots with antibodies to the K8 antibody. As shown in figure 1F, WT GFPK8 showed a migration pattern similar to endogenous K8, whereas both of the phospho-site mutants showed a migration pattern similar to that observed in the PKP3 knockdown clones. Further, both phospho-site mutants showed an increased stability as compared to WT K8. A Western blot for β-actin served as a loading control (figure 1F). Other modifications such as the levels of O-linked N-acetylglucosamine (O-GlcNAc) on K8 [38], [39], [40], were not altered in the PKP3 knockdown clones (Figure S2E). These results suggest that all the phenotypic changes observed in our experiments are probably due to alterations in phosphorylation on K8.

**Figure 2:**
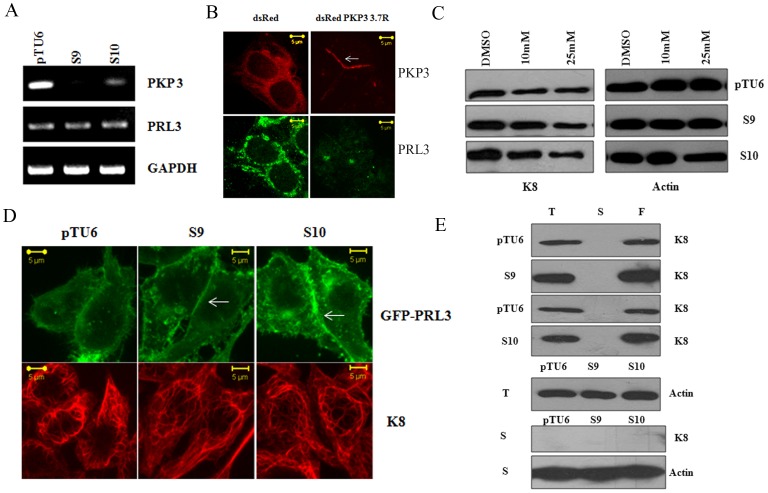
Plakophilin3 loss leads to an increase in PRL3 levels. **A.** RNA prepared from the vector control or PKP3 knockdown clones was used as a template in reverse transcriptase coupled PCR reactions to determine the mRNA levels of PRL3 and PKP3. A PCR for GAPDH served as a loading control. **B.** S9 cells were transfected with either dsRed or the shRNA resistant dsRed PKP3 3.7R cDNA. 48 hours post transfection cells were stained with antibodies to PRL3 (green) and visualized by confocal microscopy. Note that dsRed PKP3 3.7R localizes to the border as previously described (indicated by arrow) [28]. Original magnification is 630X with a 2X optical zoom. Scale bar 5 µm. **C.** The vector control or PKP3 knockdown clones were treated with either the vehicle control (DMSO) or the indicated concentrations of the PRL3 inhibitor. Protein extracts were resolved on gels followed by Western blotting with antibodies to K8 and β-actin. **D.** GFP PRL3 was transfected into either vector control (pTU6) or PKP3 knockdown clones (S9 and S10). 48 hours post transfection, the cells were stained with antibodies to K8 (red) and visualized by confocal microscopy. Note that GFP PRL3 shows a marginally enhanced localization to the border in S9 and S10 cells in contrast to pTU6 and doesn’t show increased localization on K8 filaments. Original magnification is 630X with a 2X optical zoom. Scale bar 5 µm. **E.** Total cell extracts (T), Soluble fractions (S) and the filament fractions (F) were prepared as described from either the vector control or PKP3 knockdown clones. Equal cell equivalents of these extracts were loaded on 10% SDS-PAGE gels followed by Western blotting with antibodies to K8 (top four panels). A Western blot for β-actin was performed in the total cell extracts as a loading control (fifth panel). 100 µg of soluble fractions were resolved on SDS-PAGE gels followed by Western blotting for K8 or β-actin (bottom two panels).

It has been reported previously that an increase in PRL3 levels leads to a decrease in K8 phosphorylation and an increase in K8 levels [23]. A Western blot analysis demonstrated that an increase in PRL3 levels was observed in the PKP3 knockdown clones (S9 and S10) as compared to the vector control (pTU6) (figure 1A). This decrease was not due to an increase in mRNA levels as a Reverse Transcriptase coupled polymerase chain reaction (PCR) demonstrated that PRL3 RNA levels were similar in the vector control and PKP3 knockdown clones (figure 2A). As expected PKP3 mRNA levels were lower in the PKP3 knockdown clones as compared to the vector control and GAPDH served as a loading control (figure 2A). To determine if the increase in PRL3 levels could be reversed by an shRNA resistant PKP3 cDNA, a rescue experiment was performed as described above. As shown in figure 2B, S9 cells expressing dsRed PKP3 3.7R showed a decrease in PRL3 staining compared to cells transfected with dsRed. These results suggest that loss of PKP3 leads to an increase in PRL3 protein levels.

To determine whether inhibition of PRL3 could cause a decrease in the levels of K8 in the PKP3 knockdown clones, the cells were treated with either the vehicle control or increasing concentrations of PRL3 inhibitor. The levels of K8 decreased upon inhibition of PRL3 in the vector control and PKP3 knockdown clones suggesting that an increase in phosphorylation of K8 could account for the decrease in K8 stability (figure 2C). However, incubation with the inhibitor for longer than 72 hours led to cell death (data not shown). Treatment of the cells with the PRL3 inhibitor lead to a decrease in migration in the scratch wound healing assay (Figure S3C), suggesting that alterations in phosphorylation and stability of K8 could lead to a reversal of the phenotypes observed upon PKP3 loss. To determine if the loss of PRL3 could lead to a reversal of the phenotypes observed upon PKP3 knockdown, we generated a shRNA construct that could inhibit the expression of PRL3 (Figure S3D), which was based on a previously reported sequence [41]. However, when we attempted to generate stable cell lines that expressed the shRNA construct, most of the cells died and the few that survived selection did not show a knockdown of PRL3 (Figure S3E), a result consistent with those obtained with the PRL3 inhibitor. Thus, we were unable to determine the long term consequences of PRL3 inhibition upon PKP3 knockdown.

To determine if there was a change in PRL3 localization in the cell or increased PRL3 localization on K8 filaments upon PKP3 knockdown, the vector control and knockdown cells were stained with antibodies to PRL3. As shown in Figure S3A, there is a marginal increase in PRL3 localization at the border and in a peri-nuclear compartment (arrows) in the PKP3 knockdown clones (S9 and S10) as compared to the vector control (pTU6). A similar observation was made when exogenously expressed GFP-PRL3 was transfected into the vector control and PKP3 knockdown clones (arrows, figure 2D). However, as this is accompanied by an increase in protein levels, it is hard to determine whether there is a change in localization of PRL3 or whether the increased protein levels are resulting in an accumulation at the cell border and the perinuclear compartment in the PKP3 knockdown clones.

To determine whether PRL3 shows an increased localization to K8 filaments upon PKP3 knockdown, HCT116 cells were transfected with a GFP-tagged PRL3 construct and stained with antibodies to PRL3. As shown in Figure S3B, the signal for the exogenously expressed protein overlapped with the signal generated upon immunofluorescence with the PRL3 antibodies. These results suggest that the GFP-PRL3 shows a localization similar to endogenous PRL3. As shown in figure 2D, there is no increasing the localization of PRL3 to K8 filaments in the PKP3 knockdown cells as compared to the vector control.

To determine the effects of K8 phosphorylation on filament formation, the amount of K8 in the soluble and insoluble fractions was measured in the vector control and PKP3 knockdown clones. Keratin solubility assays demonstrated that all the K8 was present in the insoluble fraction in both the vector control and PKP3 knockdown clones, even when a 100 µg of the soluble fraction was loaded (figure 2E). Further, as shown in Figure S1C, filament formation was not altered in the PKP3 knockdown clones as compared to the vector control. Similarly, staining with the phosphospecific antibodies to K8 did not result in any detectable alterations in keratin filament organization in the PKP3 knockdown clones when compared to the vector control (Figure S4A). In addition, the alteration in phosphorylation on K8 did not result in an alteration in the interaction between K8 and K18 as confirmed by Fluorescence Resonance Energy Transfer (FRET) assays (Figure S4B). These are consistent with results that suggest that the phospho-site mutants of K8 form filaments in a manner similar to WT K8 [24]. These results suggest that the change in phosphorylation does not alter either filament formation or solubility of K8.

**Figure 3:**
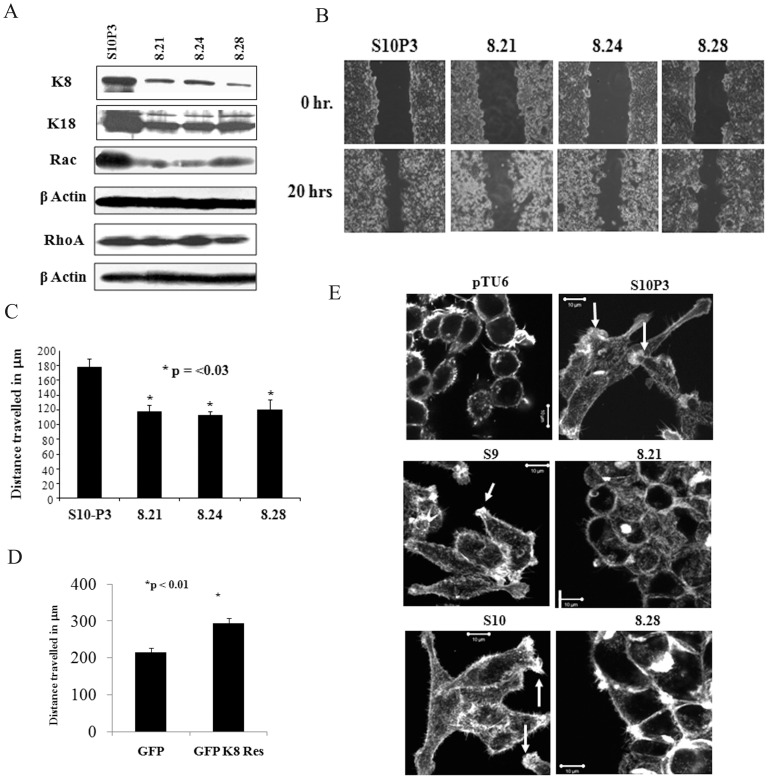
K8 knockdown leads to a decrease in migration. **A.** Protein extracts from the S10 derived K8 (8.21, 8.24 and 8.28) knockdown clones or the vector alone (S10P3) were resolved on SDSPAGE gels followed by Western blotting with antibodies to PKP3, K8, K18, rac, rhoA and β-actin. **B and C.** Scratch wound healing assays were performed on the S10 derived K8 knockdown clones or the vector control and the distance migrated measured. The data shown is the average from three independent experiments with the mean and standard error plotted as shown (* p<0.03 by students t-test). **D.** Scratch wound healing assays were performed on the double knockdown clone 8.21, transfected with either GFP alone or GFP K8 res and the distance migrated measured. The data shown is the average of three independent experiments (p<0.01 by students t-test). **E.** Scratch wound healing assays were performed on the vector control (pTU6) or PKP3 knockdown clones (S9 and S10) or the S10 derived K8 (8.21, and 8.28) knockdown clones or the vector control (S10P3). The cells were fixed and stained with FITC labeled phalloidin to visualize actin filaments, followed by confocal microscopy. Arrows indicate cells showing lamellipodia formation (Original magnification is 630X with a 2X optical zoom. Scale bar 10 µm).

To determine whether the elevated K8 levels were required for transformation upon PKP3 loss, we inhibited K8 expression using RNA interference using previously described shRNA constructs [19]. These experiments were performed in the S10 clone as previous results from the laboratory have demonstrated that the S9 and S10 clones are indistinguishable in terms of their ability to initiate desmosome formation and the transformed phenotype [28], [33]. The PKP3 knockdown clone (S10) was transfected with either the vector control or the K8 knockdown construct and single cell clones with a decrease in PKP3 and K8 expression (8.21, 8.24, 8.28) as compared to a clone transfected with the vector alone (S10P3) were identified (Figure S5A and figure 3A). As reported previously [42], [43], a decrease in the levels of K8 lead to a decrease in the levels of K18 (figure 3A), despite the fact that these constructs do not inhibit K18 [19]. Further, the level of the other type I keratin was also altered in the double knockdown clone 8.21 suggesting that it forms a complex with K8 (Figure S1B). An immunofluorescence analysis demonstrated that filament formation was not affected in the knockdown clones (Figure S5B), though the knockdown cells showed a decrease in the immunofluorescence signal as compared to the vector controls. These results show that the alteration in K8 levels does not lead to alterations in the ability of K8 to form filaments. Similarly, knockdown experiments were performed for K18 in the PKP3 knockdown clone. The knockdown of K18 did not lead to a huge decrease in the levels of K8 (Figure S5C), presumably because of the presence of another type I keratin in the cells (Figure S1B).

The PKP3 knockdown cells show a decrease in cell-cell adhesion and desmosome size and an increase in cell migration [33]. Wound healing assays demonstrated that the double knockdown clones, showed a decrease in cell migration as compared to the PKP3 knockdown cells (figures 3B and 3C). These differences were significant at multiple time points and the rate of migration was significantly different at multiple time points (Figure S5D and S5E). To determine that the results observed in HCT116 cells were not due to off target effects of the shRNA, the double knockdown clone, 8.21, was transfected with the shRNA resistant K8 construct (GFP K8 Res) or a vector expressing GFP and migration assays were performed as described. Expression of GFP K8 Res, but not GFP, resulted in increased migration in the double knockdown clones (Figure S5D and figure 3D). Similar results have been reported in other cell types [19]. Therefore, the results observed are due to a K8 knockdown and not due to off-target effects of the shRNA.

An increase in cell migration is often accompanied by an increase in actin dynamics and the formation of actin dependent structures such as lamellipodia and filopodia (reviewed in [44], [45]). To determine if an increase or decrease in K8 levels affects actin dynamics in migrating cells, wound healing assays were performed followed by staining with phalloidin-FITC to visualize actin filaments at the migrating edge. As shown in figure 3E, the PKP3 knockdown clones showed more lamellipodia formation than the vector control cells, which is consistent with the observation that migration increases in the PKP3 knockdown clones [33]. In contrast, the double knockdown clones showed a thin layer of cortical actin with the presence of small ciliary structures. Western blot data demonstrated that actin levels are similar in all clones tested (figures 1A and 3A). Previous reports have suggested that lamellipodia formation is accompanied by an increase in rac activity [46]. A Western blot analysis demonstrated that the levels of rac were decreased in the double knockdown clones as compared to the vector control, while no changes were observed in rhoA levels (figure 3A). Thus, the double knockdown clones show a decrease in the formation of lamellipodia and an alteration in actin morphology as compared to the PKP3 knockdown clone, which may be responsible for the reversion of the migration phenotype accompanying the K8 knockdown.

**Figure 4:**
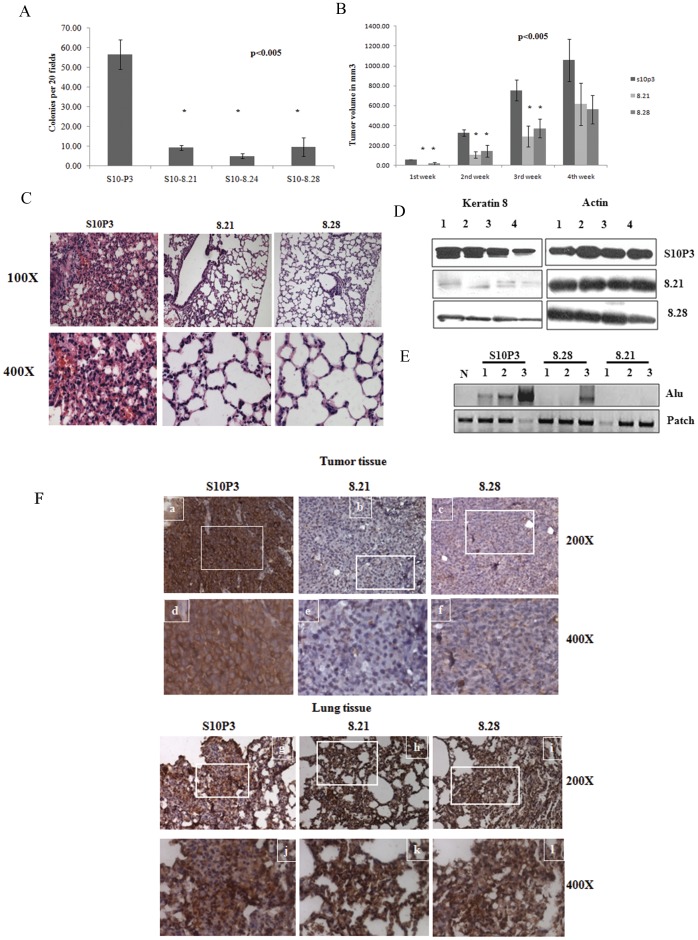
K8 downregulation leads to an inhibition of transformation in vitro and in vivo. **A.** The S10 derived K8 (8.21, 8.24 and 8.28) knockdown clones or the vector alone (S10P3) were plated in soft agar and colony formation determined after 2–3 weeks. The number of colonies formed by the clones per 20 low power fields (10X) was counted in triplicate in each experiment and the mean and standard deviation of three independent experiments is plotted as shown. **B.** 10^6^ cells from the S10 derived K8 (8.21 and 8.28) knockdown clones or the vector alone (S10P3) were injected subcutaneously into 5 different nude mice and tumor size determined every week as described. Tumor volume is plotted on the Y-axis and the time in weeks on the X-axis. **C.** Protein extracts from primary tumors from mice injected with the S10 derived K8 (8.21 and 8.28) knockdown clones or the vector alone (S10P3) were resolved on SDS-PAGE gels followed by Western blotting with antibodies to K8 and β-actin. The numbers indicate different mice injected with the single or double knockdown clones. All the samples were run on the same gel and the Western blots performed at the same time. **D.** Haematoxylin and eosin staining of paraffin embedded sections of lung tissue from nude mice injected with 10^6^ cells of the vector alone (S10P3) or the double knockdown clones (8.21 and 8.28). Lung section from mice injected with S10P3 cells show extensive metastasis with thickening of alveolar walls from deposition and aggregation of metastasized tumor cells, whereas lungs from mice injected with the double knockdown cells show normal lungs with thin walled alveoli, with a few metastatic tumor cells. The images in the top row are at magnification x100 and images in the bottom row are at magnification x 400. **E.** PCR reactions were performed on DNA isolated from paraffin sections for the presence of Alu repeats in genomic DNA. Genomic DNA was purified from normal lung tissue, lung tissue from mice injected with cells with PKP3 knockdown alone (S10P3) and lung tissue from mice injected with the double knockdown clones (8.21 and 8.28). Lung tissues from uninjected mice (N) were used as a negative control for the Alu PCR. A PCR for the mouse patch gene was performed as a loading control. **F.** Immunohistochemical staining was performed with antibody against K8 on sections of paraffin embedded tissue of tumor and lungs of mice injected with S10P3 vector control cells or the double knockdown clones, 8.21 and 8.28. Images a, b, c, g, h and i are taken at a magnification x 200 and the respective magnified images at magnification x 400, of the indicated areas in the white rectangles, are represented by images d, e, f, j, k and l. Tumor tissue from mice injected with S10P3 (a and d) show K8 staining at levels higher compared to tumors from mice injected with the double knockdown clones, 8.21 and 8.28 (b, c, e and f). In contrast lung tissue, from mice injected with the vector control S10P3 cells (g and j) and the double knockdown clones (8.21 and 8.28) (h, i, k and l) show elevated K8 staining in metastatic areas of the lungs with infiltrated tumor cells.

To determine whether K8 loss lead to a decrease in the transformed phenotype of the PKP3 knockdown clones, soft agar assays were performed. As shown in figure 4A, the double knockdown clones formed significantly fewer colonies in soft agar as compared to the PKP3 knockdown clones. In contrast, K18 knockdown did not lead to a significant decrease in colony formation in soft agar (Figure S6A). The ability of the double knockdown clones to form tumors in immunocompromised mice was monitored weekly over a period of four weeks. As shown in figure 4B, the double knockdown clones 8.21 and 8.28 formed smaller tumors as compared to the PKP3 knockdown clone. All five mice injected with the vector control and the 8.28 clone developed tumors while four mice injected with the 8.21 clone developed tumors. The difference in size was statistically significant at all the time points studied for the 8.28 clone and for the first three weeks for the 8.21 clone. These results are similar to those observed in other cell lines [19]. A Western blot analysis for tumors formed in four mice for each cell type demonstrated that tumors derived from the K8 knockdown clones have a lower level of K8 protein than those derived from the vector control (figure 4D). A Western blot for actin was performed as a loading control. A quantitation for these Western blots is shown in Figure S6B.

Previous reports from this laboratory show that a decrease in PKP3 levels leads to lung metastasis [33]. To determine if the K8 knockdown lead to a decrease in metastasis, lung sections from mice injected with either the PKP3 knockdown clones (S10P3) or the double knockdown clones (8.21 and 8.28) were examined for the presence of metastatic colonies in the lungs. As shown in figure 4C and table 1, the double knockdown clones showed a decrease in lung metastasis as compared to the PKP3 knockdown clones. More metastasis was observed in mice injected with the double knockdown clone 8.28 as compared to 8.21, which is consistent with these cells having higher K8 expression (figure 4D and Figure S6B).

Detection of Alu repeats in genomic DNA has been used previously to determine whether human cells are present in xenograft models [47]. To determine whether the infiltrating cells in the lungs were of human origin PCR reactions for Alu repeat DNA were performed. As shown in figure 4E, genomic DNA purified from normal mouse lungs was negative for Alu repeat DNA. In contrast, genomic DNA purified from lungs from the mice injected with the S10P3 clone showed the presence of the Alu repeats. Genomic DNA from mice injected with the double knockdown clones did not contain Alu repeat sequences with the exception of one animal that showed increased metastasis to the lungs. A PCR for the mouse patch gene was performed as a loading control (figure 4E). These results suggest that the infiltrating cells in the lungs are of human origin and are therefore metastatic cells derived from the primary tumor.

**Table 1:** PKP3 CK8 double knockdown clones show a decrease in lung metastasis.

	Number of mice with		
Clone	No metastasis	<30% metastasis	>30% metastasis
S10P3	0	0	5
8.21	2	2	1
8.28	0	2	3

Hematoxylin-eosin stained sections of lungs from mice injected with the indicated clones were examined for the presence of infiltrating tumor cells and the percentage of area occupied by the tumor cells determined microscopically. Five mice were tested for each clone.

To determine whether metastatic colonies formed in mice injected with the double knockdown clones had regained K8 expression, the levels of K8 were analyzed using immunohistochemistry in both the primary tumors and lung sections with infiltrating tumor cells. Primary tumors derived from the vector control (S10P3) showed high levels of K8 as compared to primary tumors derived from the double knockdown clones (8.21 and 8.28) (figure 4F). Primary tumors derived from mice injected with 8.28 showed slightly higher levels of immunoreactivity as compared to tumors derived from mice injected with the 8.21 clone, a result consistent with the Western blot data (figure 4C). However, metastatic colonies in the lungs showed equivalent expression of K8 in mice injected with all three clones (figure 4F). These results suggest that an increase in K8 expression upon PKP3 knockdown is required for tumor progression and metastasis. Therefore, it appears that metastasis to the lungs requires K8 expression as all the metatstatic colonies in the lungs showed high K8 expression.

## Discussion

Loss of the desmosomal plaque protein plakophilin3, leads to an increase in tumor progression and metastasis [33]. PKP3 loss is accompanied by an increase in K8 levels due to a decrease in K8 phosphorylation. A shRNA mediated decrease in K8 expression in the PKP3 knockdown clones leads to a decrease in migration, a decrease in colony formation in soft agar and decreased tumor size and metastasis in mice.

PKP3 loss leads to an increase in metastasis to the lungs (figure 4 and [33]). K8 loss led to a decrease in the ability of the PKP3 knockdown clone, S10 to metastasize to the lungs (figure 4 and table 1). Importantly, most of the metastatic cells observed in mice injected with the double knockdown clones reacquired K8 expression (figure 4F). The infiltrating cells are of human origin as they contain Alu repeat DNA (figure 4E). As the infiltrating cells in the lung express K8 and are of human origin, it strongly suggests that these represent a metastatic growth from the primary tumors. The decrease in tumor size and metastasis suggest that K8 is required for transformation downstream of PKP3 loss. PKP3 has been reported to form a complex with K18 (Figure S1 and [27]), and may play a role in anchoring IF’s to the desmosome leading to the creation of a tissue wide IF network and improved mechanical rigidity (reviewed in [3]). In the absence of desmosome function, it is possible that the IF network is reorganized to maintain rigidity to permit cell survival. One way to accomplish this would be to increase filament formation by increasing keratin stability.

Another possible explanation for why an increase in K8 levels leads to increased metastasis might be the effect K8 has on cell migration. Loss of K8 in the PKP3 knockdown clones results in an inhibition of migration, reversing the phenotype observed upon PKP3 loss [33]. The observed phenotypes were due to a decrease in K8 levels as expression of an shRNA resistant K8 cDNA rescued these phenotypes. K8 loss was accompanied by a decrease in lamellopodia formation and a decrease in the levels of Rac. Therefore, the increase in PRL3 levels induced by PKP3 loss might not only alter the post translational modifications on IF’s but also lead to alterations in the way IF’s communicate with the actin cytoskeleton through their effects on integrin signalling as previously reported [19].

The GFP tagged phosphosite mutants show an increase in stability in comparison to the GFP tagged WTK8. These results suggest that the increase in stability observed upon PKP3 knockdown is not due to an increase in translation, given that the WT and mutant proteins are being expressed from a heterologous promoter with different 5′ and 3′ untranslated regions from the endogenous K8 gene. This is important because plakophilin family members, including PKP3, have been shown to regulate translation [48], [49]. Treatment of the vector and PKP3 knockdown clones with a PRL3 inhibitor leads to a decrease in K8 levels in both the vector control and knockdown clones, which is consistent with our observations on the migration phenotypes of the phospho-site mutants in two-dimensional gels (figure 2). Consistent with this observation, our data demonstrates that PRL3 protein levels are increased in the PKP3 knockdown clones and this increase in expression could be reversed by over-expression of a shRNA resistant PKP3 cDNA (figure 2B). Further, other than a minor increase in localization at the cell border, no changes in PRL3 localization or the association of PRL3 with keratin filaments were observed in these studies.

PRL3 has been reported to dephosphorylate K8 at both S73 and S431 [23]. Given this data, we favour the hypothesis that PKP3 loss leads to a decrease in the levels of a kinase that phosphorylates S431 and an increase in PRL3 levels (figures 1 and 2) due to either an alteration in signalling events from the desmosome [50] or due to the regulation of translation of PRL3 by PKP3, as PKP3 has been shown to regulate translation [48]. The increase in PRL3 levels in the PKP3 knockdown clones happens post-transcriptionally, as PRL3 mRNA levels do not change in the PKP3 knockdown clones as compared to the vector controls, but protein levels do go up. Therefore, it is possible that PKP3 regulates the translation of PRL3 with subsequent effects on K8 phosphorylation and stability. Alternatively, PKP3 could positively regulate the expression of Poly C RNA Binding Protein 1 (PCBP1), which has been shown to inhibit PRL3 translation [51].

It has been reported previously that phosphorylation of keratins leads to an increase in solubility and filament network disassembly [37], while dephosphorylation leads to an increased filament formation and an increase in the insoluble fraction. However, we do not observe any changes in filament formation and solubility upon PKP3 knockdown and the subsequent dephoshphorylation of K8 in HCT116 cells. This could be due to cell type/tissue type specific effects. Consistent with this hypothesis, Mizuuchi et al. have not observed any changes in keratin8 organization upon expression of GFP-tagged PRL-3 in SW480 cells derived from the colon [23]. Therefore, it is possible that in cell lines derived from the colon, changes in K8 phosphorylation do not lead to changes in filament formation or solubility. Similarly, no change in the localization of phosphorylated K8 (Figure S4A) or the interaction between K8 and K18 (Figure S4B) were observed in the PKP3 knockdown cells suggesting that loss of PKP3 does not affect organization of the IF cytoskeleton.

The requirement for an intact filament network for transformation is supported by the data that knockdown of K18 does not phenocopy K8 knockdown, which might be due to the fact that another type I keratin is present in these cells (Figure S1B). The increase in these biochemical properties is also accompanied by an increase in transformation potential, as observed upon an increase in PRL3 expression [23]. In agreement with these previously published observations, an increase in PRL3 expression was observed upon PKP3 knockdown and inhibition of PRL3 activity lead to a decrease in K8 levels in the experiments described in this report. We were unable to determine if the chemical inhibitor of PRL3 could inhibit transformation as incubation of these cells with the PRL3 inhibitor for greater than 72 hours led to cell death (data not shown) and we were unable to generate clones with a double knockdown of PKP3 and PRL3 (Figure S3D). Further, another report from our laboratories demonstrates that dephosphorylation of K8 correlates with increased metastasis in human oral squamous cell carcinoma and leads to increased transformation in vivo and in vitro [24]. Taken together these results suggest that alterations in K8 phosphorylation could lead to tumor progression and increased metastasis.

The results described herein suggest that PKP3 loss leads to alterations in phosphorylation on K8, due to an increase in PRL3 levels. These result in increased K8 levels which promote increased migration, transformation and metastasis. Loss of K8 in the PKP3 knockdown clones leads to a reversal of these phenotypes, suggesting that an increase in K8 levels is required for the neoplastic progression and metastasis induced upon PKP3 loss. These results in conjunction with our data on the levels of phosphorylated keratin in human oral squamous cell carcinoma [24] suggest that post translational modifications on keratins could be important predictors of metastatic disease.

## Materials and Methods

### Ethics Statement

Maintenance of the animal facility is as per the national guidelines provided by the Committee for the Purpose of Control and Supervision of the Experiments on Animals (CPCSEA), Ministry of Environment and Forest, Government of India. The animals were housed in a controlled environment with the temperature and relative humidity being maintained at 23±2°C and 40–70% respectively and a day night cycle of 12 hrs each (7∶00 to 19∶00 light; 19∶00 to 7∶00 dark). The animals were received an autoclaved balanced diet prepared in-house as per the standard formula and sterile water *ad libitum*. Mice were housed in the Individually Ventilated Cage (IVC) system (M/S Citizen, India) provided with autoclaved rice husk bedding material available locally. Protocols for the experiments were approved by the Institutional Animal Ethics Committee (IAEC) of the Advanced Centre for Treatment Reserach and Education in Cancer (ACTREC). The title of the project is “Mechanisms underlying tumor progression upon PKP3 loss in epithelial cells.” The project approval number is 13/2007 and approval was granted on the 27^th^ of July, 2007.

### Plasmids and Constructs

The cloning of K8 shRNA constructs and the shRNA resistant K8 (GFP K8 Res) has been described elsewhere [19]. The phosphosite mutants S73A and S431A [37], [52] were a kind gift of Dr. Normand Marceau and were cloned downstream of GFP in a manner similar to the WT K8 construct. To generate plasmid based shRNA constructs targeting PRL-3, the oligonucleotide pairs listed in table 2 were cloned into Age1-EcoR1 digested pLVU6 plasmid, downstream of the U6 promoter. To generate GFP tagged PRL-3, PRL-3 was PCR amplified from eukaryotic cDNA library as a BamH1-Xho1 fragment and cloned into EGFP-f N1 (Clontech), digested with BglII-Xho1, which resulted in the deletion of the farnesylation signal.

**Table 2:** Oligonucleotides used for cloning, genomic PCRs and reverse transcriptase PCR reactions.

Construct	Oligonucleotides
K8.1	5′ ccggcagcagcaactttcgcggtaagttctctaccgcgaaagttgctgctgccttttttc 3′ and 5′ tcgagaaaaaaggcagcagcaactttcgcggtagagaacttaccgcgaaagttgctgctg 3′
K8.2	5′ ccggcatcaccgcagttacggtcaaagttctcttgaccgtaactgcggtgatgccttttttc3′ and 5′ tcgagaaaaaaggcatcaccgcagttacggtcaagagaactttgaccgtaactgcggtgatg 3′
K8.3	5′ ccggagagctggccattaaggataagttctctatccttaatggccagctctccttttttc 3′ and 5′ tcgagaaaaaaggagagctggccattaaggatagagaacttatccttaatggccagctct ′3
Alu 5′	5′ acg cct gta atc cca gca ctt 3′
Alu 3′	5′ tcg ccc agg ctg gag tgc a 3′
Ptc 5′	5′ ctgcggcaagtttttggttg 3′
Ptc 3′	5′ agggcttctcgttggctacaag 3′
PRL3 FWD	5′ gagctacaaacacatgcgc 3′
PRL3 REV	5′ gaacttggccttcaccaggctca 3′
PRL3shRNA a	5′ccggaacagcaagcagctcacctacctcgaggtaggtgagctgcttgctgtttttttg3′
PRL3shRNA b	5′aattcaaaaaaacagcaagcagctcacctacctcgaggtaggtgagctgcttgctgtt3′

### Cell Lines and Transfections

HCT116 cells, an epithelial cell line derived from a human colorectal carcinoma, were obtained from Dr. Bert Vogelstein ([53] and ATCC). The HCT116 derived PKP3 knockdown clones were cultured as described previously [33]. Cells were transfected by calcium phosphate precipitation protocol as described [54]. To generate K8 PKP3 double knockdown clones, the previously described HCT116 derived plakophilin3 knockdown clone S10 [28], [33], was transfected with 3 µg of the K8.2 shRNA construct and selected in medium containing 0.5 µg/ml of puromycin (Sigma) to generate single-cell clones. To determine the contribution of K8 phosphorylation to K8 stability, 3 µg of WT K8 or the phosphosite mutants were transfected into HCT116 cells in a 35 mm dish as described [33]. To perform the rescue experiments, 3 µg of EGFPN1 (Clonetech) or GFP K8 Res were transfected into the double knockdown clone. 48 hours post transfection the cells were processed for cell-cell adhesion assays and for wound healing assays as described below. The PRL3 inhibitor (Sigma P0108) dissolved in DMSO was added to cells for 48 hours before extracts were prepared for Western blotting.

### High Salt Keratin Extraction

The high salt extraction for keratins was performed as previously described [40]. Briefly, HCT 116 cells were trypsinized and lysed in detergent buffer (10 mM Tris pH 7.6, 140 mM Nacl, 5 mM EDTA, 1% Triton X-100) with protease inhibitors and incubated for 30 minutes on ice. The lysates were cleared by centrifugation and suspended in high salt buffer (10 mM Tris pH 7.6, 140 mM Nacl, 1.5 M KCl 0.5% TritonX-100) with protease inhibitors and stirred for 1–3 hours at 4°C. The insoluble material was washed thrice in 10 mM Tris pH 7.6, the pellet suspended in 2% SDS and 50 µg of protein loaded on SDS-PAGE gels followed by staining with coomassie brilliant blue.

### Antibodies, Western Blotting and Solubility Assays

The primary antibodies for PKP3 (clone 23E3-4, Zymed, dilution 1∶1000), β Actin (mouse monoclonal, Sigma, dilution 1∶5000), K8 (Sigma, dilution 1∶5000), K18 (Sigma, dilution 1∶5000), phosphospecific antibodies for K8 Serine 73 (mouse monoclonal Lifespan Biosciences, dilution 1∶2000) or Serine 431 (rabbit polyclonal Abcam, dilution 1∶5,000), p38 (Santa Cruz, dilution 1∶500), rac (Cell Signalling 1∶500) rhoA (Cell Signalling 1∶500) and PRL3 (Abcam 1∶500) were used for Western blot analysis. Respective secondary antibodies were used at a dilution of 1∶1000 (Invitrogen) or 1∶5000 (Pierce). Protein samples were resolved on a polyacrylamide gel by SDS PAGE and then transferred to a nitro cellulose or PVDF membranes and processed for Western blot analysis as described [55]. Keratin solubility assays were performed as described [56], except that the soluble fraction was obtained by lysing the cells in 1% NP-40 in 1 X PBS and the 1% NP-40 insoluble fraction was dissolved in 1% SDS.

### FRET Assays

The HCT116-derived PKP3 knockdown clone S9 and S10 and the vector control were grown on glass bottom plates and co-transfected with constructs expressing YFP K18 and CFP K8. 24 hours post transfection the cells were fed with DMEM lacking phenol red and placed on ice for 10 minutes prior to FRET to minimize mobility of the cells. FRET measurements were done using the acceptor photobleaching method in live cells [57]. FRET Calculations were performed using the following formula.

FRET _efficiency_ = (D_post_−B_post_)−(D_pre_−B_pre_)/(D_post_−B_post_).

The nomenclature and equations for FRET calculations and the FRET protocol was obtained from the Centre for Optical Instrumentation Laboratory, Wellcome Trust Center for Cell Biology, University of Edinburgh.

### Wound Healing Assays

Wound healing assays were performed as previously described [33]. Cells were observed by time lapse microscopy and images were taken every 5 minutes for 20 hours using an AxioCam MRm camera with a 10 X phase 1 objective. Migration was measured using Axiovision software version 4.5 (Zeiss). Similar experiments were performed to compare the migratory properties of PKP3 K8 double knockdown clones transfected with either GFP or GFP K8 Res.

### Immunofluorescence and Confocal Microscopy

Staining for K8 was performed as described [18] using the K8 monoclonal antibody (Sigma, dilution 1∶200). Filamentous actin was visualized by staining with a FITC-phalloidin conjugate (Sigma, dilution 1∶20) according to the manufacturer’s instructions. Staining for PRL3 was performed using PRL3 antibody (Abcam, dilution1∶50). Confocal images were obtained with a LSM 510 Meta Carl Zeiss Confocal system with an Argon 488 nm and Helium/Neon 543 nm lasers. All images were obtained using an Axio Observer Z.1 microscope (numerical aperture [NA] 1.4) at a magnification of X 630 with 2X optical zoom.

### Soft Agar Assays

The knockdown clones were trypsinized and counted. 2,500 cells were plated in 0.4% soft agarose and the cells were maintained in the presence of the relevant antibiotic as previously described [58]. After 2–3 weeks, the number of colonies was counted in triplicate. Three independent experiments were performed for each clone analyzed.

### Tumor Formation in Nude Mice

Nude mice experiments were performed as described [33]. All animal studies were approved by the Institutional Animal Ethics committee constituted under the guidelines of the CPCSEA, Government of India. 10^6^ cells of the HCT116 derived PKP3 knockdown or K8 PKP3 double knockdown clones were resuspended in DMEM medium without serum and injected subcutaneously in the dorsal flank of 6–8 weeks old NMRI Nude (Nu/Nu) [59] (obtained from ACTREC animal house facility). Five mice were injected for each clone. Tumor formation was monitored at intervals of 2–3 days and tumor size was measured by a vernier calipers. Tumor volume (mm^3^) was calculated by the formula ½ LV^2^ where L is the largest dimension and V its perpendicular dimension, as previously reported [59], [60].

### Histology and Immunohistochemistry

Four weeks after subcutaneous injection, the mice were sacrificed and the tumor and lung tissues were fixed in 10% formaldehyde (SIGMA) overnight and processed for histology. Five micron sections of paraffin embedded tissue were prepared and hematoxylin/eosin staining and immunohistochemical staining was performed according to standard methods [61]. The degree of metastasis was determined by examining three lung sections per mouse and visualizing the area of alveolar tissue that was filled with metastatic colonies. Permeabilization for antigen retrieval was performed by microwaving the fixed tissue sections in 10 mM Tris buffer (pH 9) with 2 mM EDTA.

### Genomic PCR for Identification of Alu Repeats

Genomic DNA was purified from paraffin sections as previously described [62]. 100ng and 200ng DNA were used as templates for amplification of the patch and Alu products respectively. The oligonucleotides used to amplify the Alu repeats and the patch gene are listed in table 2. Polymerase chain reactions for Alu repeats were performed as previously described [47].

### Reverse Transcriptase and Real Time PCR Analysis

Total RNA was extracted from the HCT116 derived vector control (pTU6) and PKP3 knockdown clones (S9, S10 and S17) using Qiagen RNeasy kit and reverse transcribed using the Omniscript RT kit (Qiagen) as per the manufacturers instructions. PCR reactions for PKP3, PRL3 and GAPDH were performed on the cDNA using Taq polymerase (NEB) as per the manufacturers instructions. Real time PCR analysis using TaqMan gene expression assays (Applied Biosystems) were performed using gene specific probes for K8 and K18 from Applied Biosystems. A similar assay for GAPDH was performed to serve as an internal control for normalization. Fold changes in expression of K8 and K18 in the plakophilin3 knockdown clones were calculated relative to the expression levels in the pTU6 vector control cells as described previously [63], [64].

### Two Dimensional Gel Electorphoresis

Protein extracts were resolved on a ReadyStrip IPG strip (BioRad) pH range 5–8 according to the instructions supplied by the manufacturer. The proteins were then resolved in the second dimension on 10% SDS-PAGE gels followed by Western blotting with antibodies to K8.

### Immunoprecipitations and Glycosylation Assays

To detect the interaction between K18 and plakophilin3, HCT-116 cells were lysed with EBC buffer (50 mM Tris HCL PH 8.0, 125 mM NaCl, 0.5% NP40 with protease inhibitor cocktail, 1 mM Na3Vo4, 50 mM Na-F ) and lysates were immunoprecipitated with anti K18 antibody. The immunoprecipitates were washed with NET-N buffer (20 mM Tris-HCL PH 8.0, 100 mM NaCl, EDTA PH 8.0, 0.5% NP40) for 3 times and run on SDS polyacrylamide gel electrophoresis followed by Western blots with antibodies to PKP3 and K18. The levels of O-linked N-acetylglucosamine (O-GlcNAc) on K8 were determined as previously described [40]. K8 was immunoprecipitated from Empigen lysates prepared from the vector control and PKP3 knockdown clones. The reactions were resolved on SDS PAGE followed by Western blots with antibodies to K8 or O-GlcNAc (ABR 1∶3000).

### Statistical Analysis

Error bars represent standard error. Statistical analysis was performed using two tailed students *t* test.

## Supporting Information

Figure S1 **K8 and K18 organization in PKP3 knockdown cells.** A. K18 forms a complex with PKP3 in HCT116 cells. EBC extracts prepared from HCT116 cells were incubated with a control antibody (IgG) or antibodies to K18 (K18). The reactions were resolved on SDS-PAGE gels and Western blots performed with antibodies to PKP3 and K18. (WCE = Whole cell extract. IP = Immunoprecipitation) B. K8 and K18 levels are altered upon PKP3 knockdown. High salt extracts from the vector control (pTU6) and PKP3 knockdown clones (S9 and S10) and the PKP3 and K8 double knockdown clone (8.21) were resolved on SDS-PAGE gels and the gel stained with coomassie blue to identify keratin bands. C. K8 and K18 form filaments in the PKP3 knockdown clones. The PKP3 knockdown clones or the vector control, were stained with antibodies against K8 or K18 (Original magnification x 630 with 2X optical zoom. Scale bar 5 µm). D. Vimentin levels are not altered upon PKP3 knockdown. Protein extracts from the vector control (pTU6), the PKP3 knockdown clones (S9, S10 and S10P3) and the double knockdown clones (8.21, 8.24 and 8.28) were resolved on SDS-PAGE gels followed by Western blotting with antibodies to vimentin. None of the clones showed the presence of vimentin. AW13516 cells served as a positive control and a Western blot for actin served as a loading control.
(TIF)

Figure S2 **Post-translational modifications on K8 and K18 upon PKP3 knockdown.** A. Migration of K18 in two-dimensional gel electrophoresis. Protein extracts from the vector control and PKP3 knockdown clones were resolved in two dimensional gels followed by Western blotting with antibodies to K18. Note that the levels of K18 are higher in the PKP3 knockdown clones but no difference in migration in two-dimensional gel electrophoresis was observed for K18. B. Densitometric analysis of K8 and phospho-K8 expression in the vector control and PKP3 knockdown clones. Expression of K8, phosphor-S73 and phosphor-S431 was normalized to that of β-actin and densitometry performed on Western blots using ImageJ software. The values for these are shown in the vector control (pTU6) and PKP3 knockdown clones (S9 and S10) as indicated. C. Testing the specificity of K8 phosphospecific antibodies. GFP tagged WTK8 or the phosphomutants (S73A and S431A) were transfected into HCT116 cells. 48 hours post transfection, protein extracts were resolved on SDS PAGE gels followed by Western blotting with either antibodies that recognize K8 or the phosphor-specific antibodies. Note that the phospho-specific antibodies do not recognize the phosphor-mutant constructs. D. p38 levels are not altered in the PKP3 knockdown clones. Protein extracts from the PKP3 knockdown clones or the vector control were resolved on SDSPAGE gels followed by Western blotting with antibodies to p38. Western blots for 14-3-3γ were performed as a loading control. E. O-linked glycosylation on K8 is not altered in the PKP3 knockdown clones. Protein extracts from the PKP3 knockdown clones or the vector control were immunoprecipitated with antibodies to K8 followed by Western blots with antibodies recognizing O-GlcNAc glycosylation or K8.
(TIF)

Figure S3 **PRL3 localization in PKP3 knockdown cells.** A. PRL3 localization in PKP3 knockdown cells. The vector control (pTU6) or PKP3 knockdown clones (S9 and S10) were stained with antibodies to PRL3 and imaged by confocal microscopy. Note that PRL3 levels increase in the PKP3 knockdown clones with an increase in perinuclear localization and a slight increase in staining at the cell border. (Original magnification x 630 with 2X optical zoom. Scale bar 5 µm). B. GFP-PRL3 colocalizes with endogenous PRL3. HCT116 cells were transfected with GFP-PRL3. The cells were fixed and stained with antibodies to PRL-3 and visualized by confocal microscopy. Note that the GFP-PRL3 signal overlaps with the endogenous PRL3 signal. (Original magnification x 630 with 2X optical zoom. Scale bar 5 µm). C. Inhibition of PRL3 results in a decrease in migration. Scratch wound healing assays were performed in the absence (dark grey bars) or presence (light grey bars) of a PRL3 inhibitor. The distance migrated is graphed on the Y-axis. The bar represents an average of three different experiments and the bars represent the standard deviation. Note that PRL3 inhibition resulted in a significant decrease in migration in all cell types. Statistical analysis was performed using two tailed students *t* test. D. Identification of an shRNA that inhibits PRL3 expression. HEK293 cells were transfected with GFP-PRL3 with the vector control or a vector expressing the PRL3 shRNA. Extracts from untransfected HEK293 cells served as controls. 60 hours post transfection, protein extracts were resolved on SDS-PAGE gels followed with Western blots with antibodies to GFP. Note that GFP-PRL3 expression is observed in the vector control but not in cells transfected with the shRNA expressing vector. Western blots for actin served as a loading control. E. Identification of PKP3 and PRL3 double knockdown clones. The PKP3 knockdown clone S10, was transfected with either the vector control (vector) or a construct expressing an shRNA targeting PRL3 (PRL3 KD). Stable clones were generated followed by Western blotting with antibodies to PRL3. Note that PRL3 levels were comparable between the cells transfected with the vector control and the PRL3 KD construct. Western blots for β-actin served as a loading control.
(TIF)

Figure S4 **K8 and K18 filament formation in PKP3 knockdown clones.** A. Localization of phosphorylated K8 in PKP3 knockdown clones. The vector control (pTU6) or PKP3 knockdown clones (S9 and S10) were stained with phospho-specific antibodies to PRL3 (S73 and S431) and imaged by confocal microscopy. Note that the intensity of the signal for pS73 increases due to an increase in K8 levels in the PKP3 knockdown clones while the intensity of the signal for pS431 remains the same in the PKP3 knockdown clones. (Original magnification x 630 with 2X optical zoom. Scale bar 5 µm). B. The interaction between K8 and K18 is not altered upon PKP3 knockdown. The vector control (pTU6) or PKP3 knockdown clones (S9 and S10) were transfected with YFP-K18 and CFP-K8. 48 hours post transfection, FRET experiments were performed as described. The region in the white rectangle was bleached in the YFP channel and a corresponding increase was observed in the CFP channel as expected. FRET efficiencies were calculated for 5 different regions in three cells and results plotted in a bar graph. The FRET efficiency for the K18 K8 pair is comparable between the vector control and the PKP3 knockdown clones as shown in the graph.
(TIF)

Figure S5 **Characterization of K8 and PKP3 double knockdown clones.** A. Generation of PKP3 and K8 double knockdown clones. Protein extracts from the S10 derived K8 (8.21, 8.24 and 8.28) knockdown clones or the vector alone (S10P3) or the vector control (pTU6) were resolved on SDSPAGE gels followed by Western blotting with antibodies to PKP3 and β-actin. B. The PKP3 K8 double knockdown clones or the PKP3 knockdown clone, were stained with antibodies against K8 (Original magnification x 630 with 2X optical zoom. Scale bars 5 µm). C. Protein extracts from the S10 derived K8 (8.21 and 8.28) or K18 knockdown clones (18.22 and 18.23) or the vector alone (S10P3) were resolved on SDSPAGE gels followed by Western blotting with antibodies to K8, K18, and β-actin. Note that K18 levels are low in the K8 knockdown clones while K8 levels are not altered substantially in the K18 knockdown clones. D. Scratch wound healing assays were performed on the double knockdown clones (8.21, 8.24 and 8.28) or the vector alone (S10P3). At different time points the distance migrated was measured and plotted as shown. The bars represent the mean of three independent experiments and the error bars represent the standard deviation. Statistical analysis was performed using two tailed students *t* test. Note that the distance migrated is significantly lowered in the double knockdown clones at 10, 15 and 20 hour time points. E. Scratch wound healing assays were performed on the double knockdown clones (8.21, 8.24 and 8.28) or the vector alone (S10P3). At different time points the distance migrated per minute was measured and plotted as shown. The bars represent the mean of three independent experiments and the error bars represent the standard deviation. Statistical analysis was performed using two tailed students *t* test. Note that the distance migrated is significantly lowered in the double knockdown clones at 10, 15 and 20 hour time points. F. Scratch wound healing assays were performed on the double knockdown clones 8.21, transfected with either GFP alone or GFP K8 res.
(TIF)

Figure S6 **Transformation induced upon keratin loss in the PKP3 knockdown clones.** A. The S10 derived K18 (18.22 and 18.23) knockdown clones or the vector alone (S10P3) were plated in soft agar and colony formation determined after 2–3 weeks. The number of colonies formed by the clones per 20 low power fields (10X) was counted in triplicate in each experiment and the mean and standard deviation of three independent experiments is plotted as shown. B. Densitometric analysis of K8 in tumor samples. Protein extracts derived from tumors generated upon injection of either the vector control (S10P3) or the double knockdown clones 8.21 and 8.28) were resolved on SDS PAGE gels followed by Western blotting Expression of K8 was normalized to that of β-actin and densitometry performed on Western blots using ImageJ software. Note that K8 levels are lower in the double knockdown clones when compared to the vector control.
(TIF)
