# Supplementary figures and images for: Plakophilin3 Loss Leads to an Increase in PRL3 Levels Promoting K8 Dephosphorylation, Which Is Required for Transformation and Metastasis
Source: PLoS One. 2012 Jun 6;7(6):e38561. doi: 10.1371/journal.pone.0038561 (PMC3368841; doi:10.1371/journal.pone.0038561)

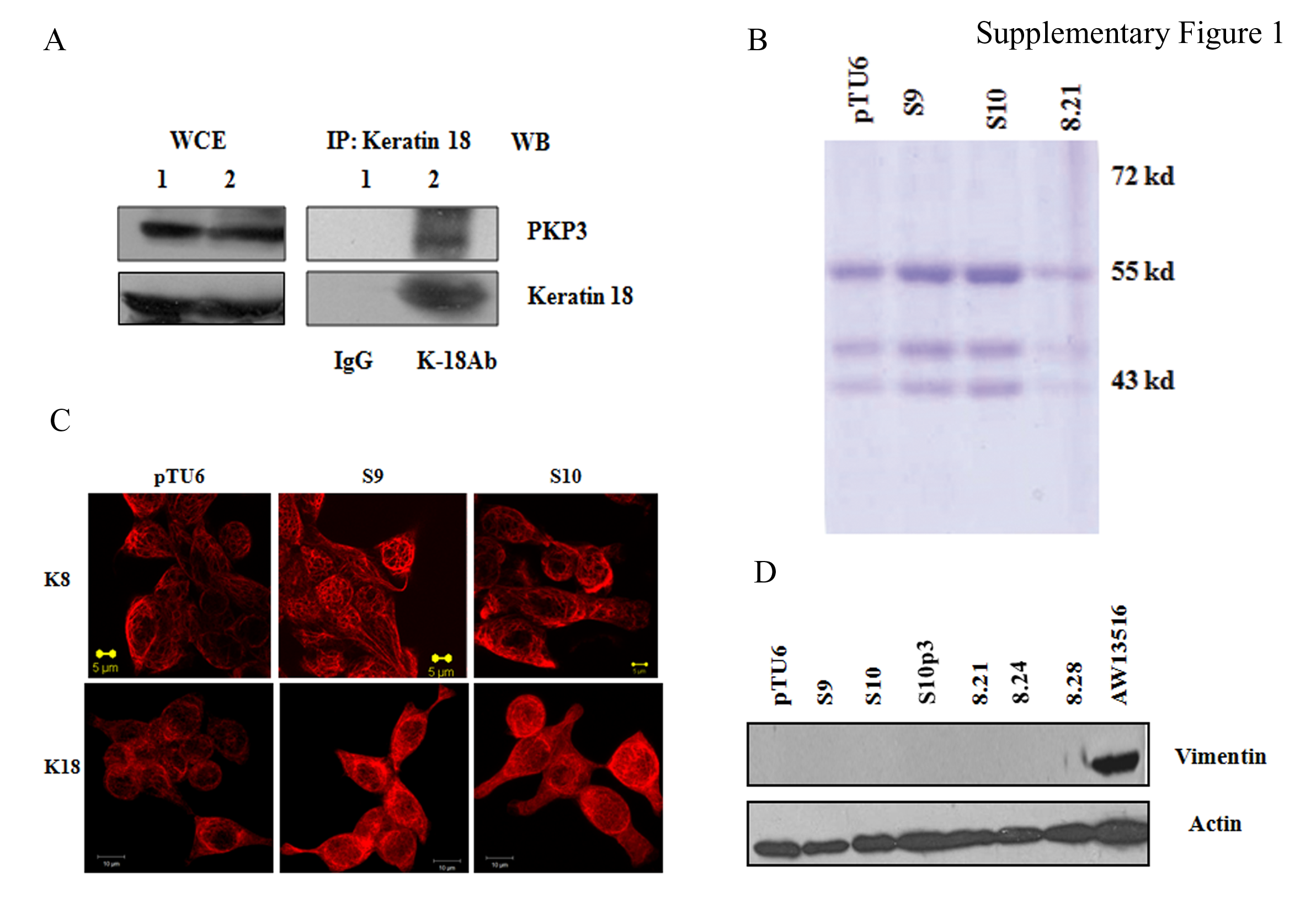

Supplement: Figure S1 — K8 and K18 organization in PKP3 knockdown cells. A. K18 forms a complex with PKP3 in HCT116 cells. EBC extracts prepared from HCT116 cells were incubated with a control antibody (IgG) or antibodies to K18 (K18). The reactions were resolved on SDS-PAGE gels and Western blots performed with antibodies to PKP3 and K18. (WCE = Whole cell extract. IP = Immunoprecipitation) B. K8 and K18 levels are altered upon PKP3 knockdown. High salt extracts from the vector control (pTU6) and PKP3 knockdown clones (S9 and S10) and the PKP3 and K8 double knockdown clone (8.21) were resolved on SDS-PAGE gels and the gel stained with coomassie blue to identify keratin bands. C. K8 and K18 form filaments in the PKP3 knockdown clones. The PKP3 knockdown clones or the vector control, were stained with antibodies against K8 or K18 (Original magnification x 630 with 2X optical zoom. Scale bar 5 µm). D. Vimentin levels are not altered upon PKP3 knockdown. Protein extracts from the vector control (pTU6), the PKP3 knockdown clones (S9, S10 and S10P3) and the double knockdown clones (8.21, 8.24 and 8.28) were resolved on SDS-PAGE gels followed by Western blotting with antibodies to vimentin. None of the clones showed the presence of vimentin. AW13516 cells served as a positive control and a Western blot for actin served as a loading control. (TIF) [file pone.0038561.s001.tif]

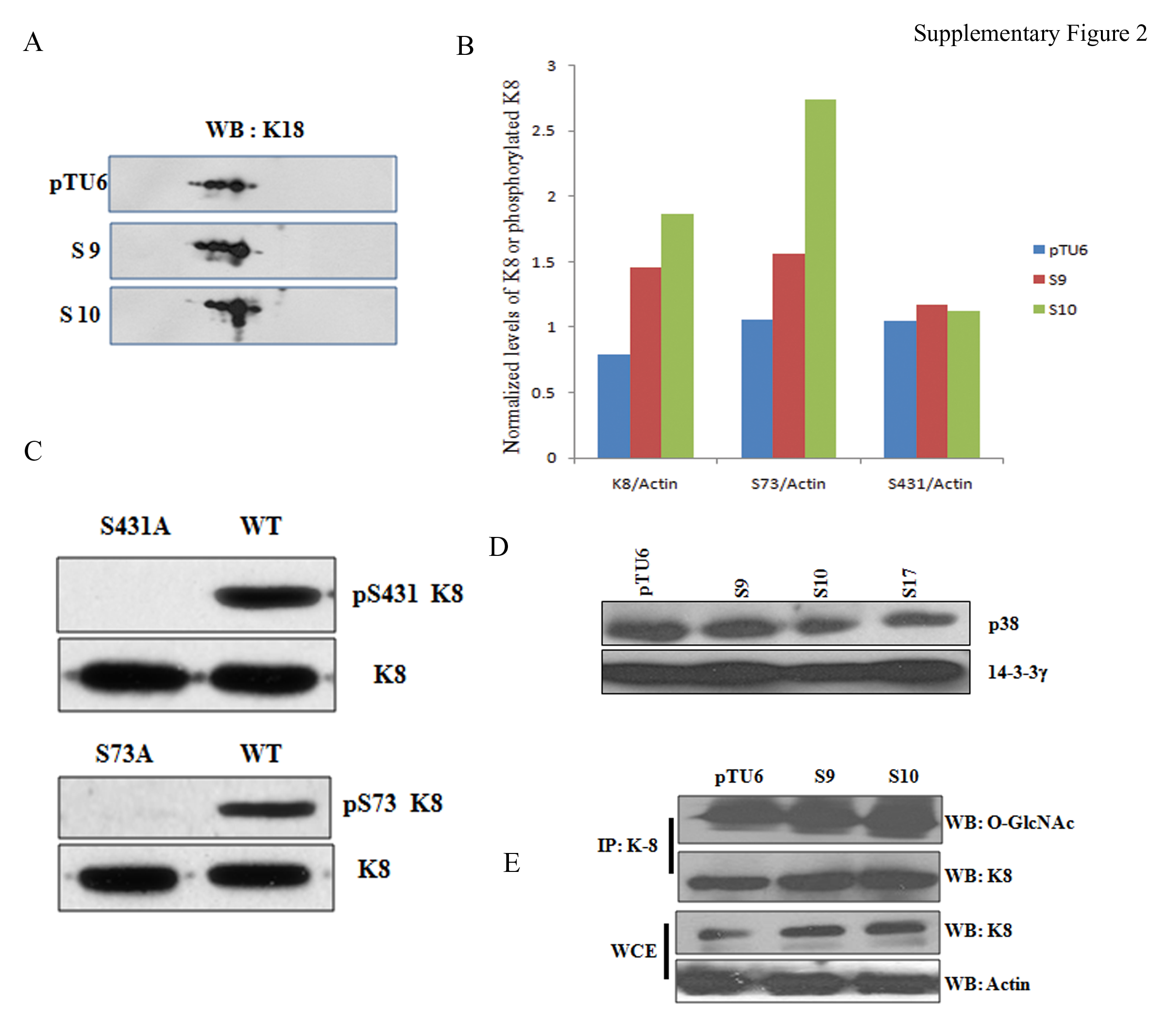

Supplement: Figure S2 — Post-translational modifications on K8 and K18 upon PKP3 knockdown. A. Migration of K18 in two-dimensional gel electrophoresis. Protein extracts from the vector control and PKP3 knockdown clones were resolved in two dimensional gels followed by Western blotting with antibodies to K18. Note that the levels of K18 are higher in the PKP3 knockdown clones but no difference in migration in two-dimensional gel electrophoresis was observed for K18. B. Densitometric analysis of K8 and phospho-K8 expression in the vector control and PKP3 knockdown clones. Expression of K8, phosphor-S73 and phosphor-S431 was normalized to that of β-actin and densitometry performed on Western blots using ImageJ software. The values for these are shown in the vector control (pTU6) and PKP3 knockdown clones (S9 and S10) as indicated. C. Testing the specificity of K8 phosphospecific antibodies. GFP tagged WTK8 or the phosphomutants (S73A and S431A) were transfected into HCT116 cells. 48 hours post transfection, protein extracts were resolved on SDS PAGE gels followed by Western blotting with either antibodies that recognize K8 or the phosphor-specific antibodies. Note that the phospho-specific antibodies do not recognize the phosphor-mutant constructs. D. p38 levels are not altered in the PKP3 knockdown clones. Protein extracts from the PKP3 knockdown clones or the vector control were resolved on SDSPAGE gels followed by Western blotting with antibodies to p38. Western blots for 14-3-3γ were performed as a loading control. E. O-linked glycosylation on K8 is not altered in the PKP3 knockdown clones. Protein extracts from the PKP3 knockdown clones or the vector control were immunoprecipitated with antibodies to K8 followed by Western blots with antibodies recognizing O-GlcNAc glycosylation or K8. (TIF) [file pone.0038561.s002.tif]

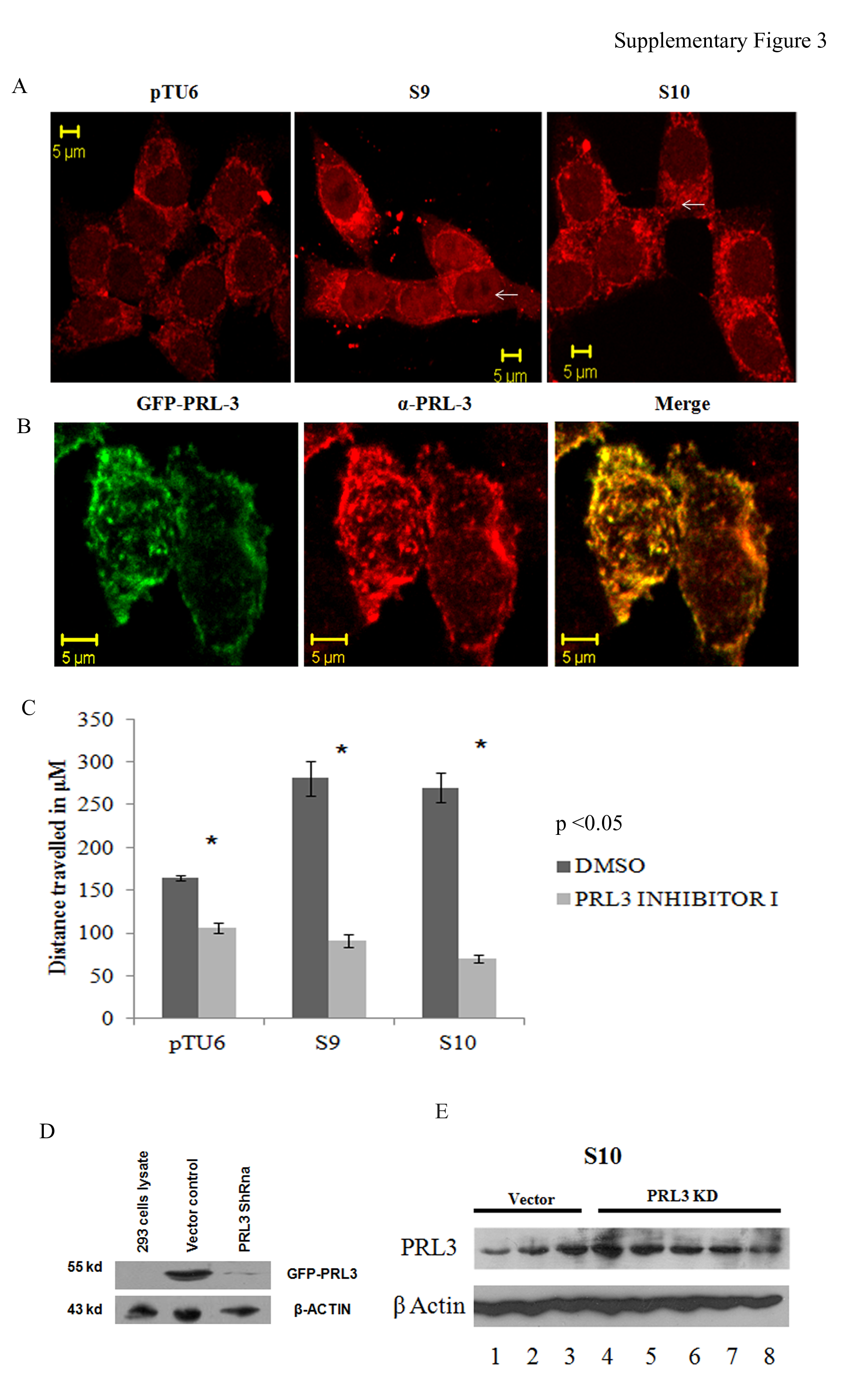

Supplement: Figure S3 — PRL3 localization in PKP3 knockdown cells. A. PRL3 localization in PKP3 knockdown cells. The vector control (pTU6) or PKP3 knockdown clones (S9 and S10) were stained with antibodies to PRL3 and imaged by confocal microscopy. Note that PRL3 levels increase in the PKP3 knockdown clones with an increase in perinuclear localization and a slight increase in staining at the cell border. (Original magnification x 630 with 2X optical zoom. Scale bar 5 µm). B. GFP-PRL3 colocalizes with endogenous PRL3. HCT116 cells were transfected with GFP-PRL3. The cells were fixed and stained with antibodies to PRL-3 and visualized by confocal microscopy. Note that the GFP-PRL3 signal overlaps with the endogenous PRL3 signal. (Original magnification x 630 with 2X optical zoom. Scale bar 5 µm). C. Inhibition of PRL3 results in a decrease in migration. Scratch wound healing assays were performed in the absence (dark grey bars) or presence (light grey bars) of a PRL3 inhibitor. The distance migrated is graphed on the Y-axis. The bar represents an average of three different experiments and the bars represent the standard deviation. Note that PRL3 inhibition resulted in a significant decrease in migration in all cell types. Statistical analysis was performed using two tailed students t test. D. Identification of an shRNA that inhibits PRL3 expression. HEK293 cells were transfected with GFP-PRL3 with the vector control or a vector expressing the PRL3 shRNA. Extracts from untransfected HEK293 cells served as controls. 60 hours post transfection, protein extracts were resolved on SDS-PAGE gels followed with Western blots with antibodies to GFP. Note that GFP-PRL3 expression is observed in the vector control but not in cells transfected with the shRNA expressing vector. Western blots for actin served as a loading control. E. Identification of PKP3 and PRL3 double knockdown clones. The PKP3 knockdown clone S10, was transfected with either the vector control (vector) or a construct expressing an shR [file pone.0038561.s003.tif]

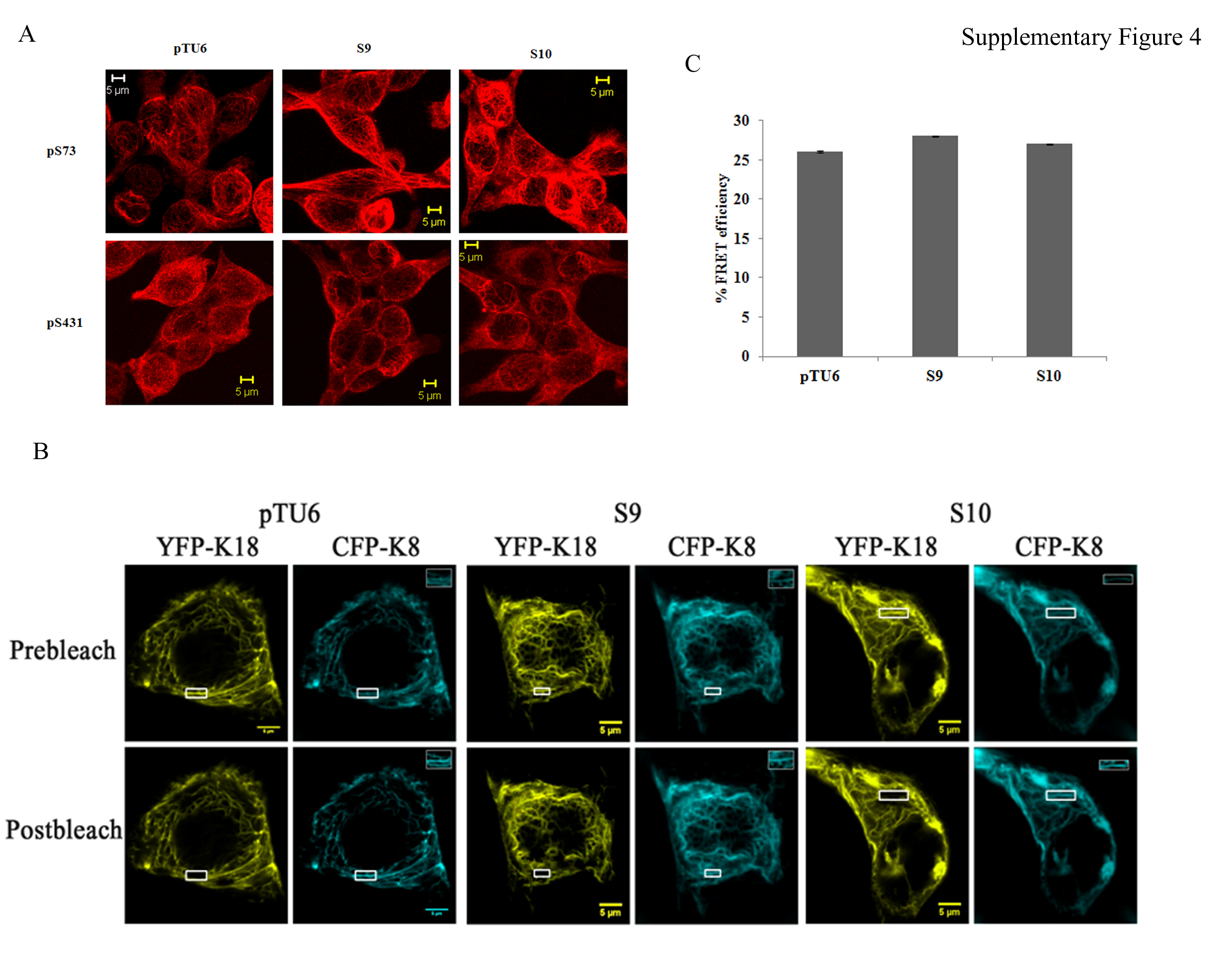

Supplement: Figure S4 — K8 and K18 filament formation in PKP3 knockdown clones. A. Localization of phosphorylated K8 in PKP3 knockdown clones. The vector control (pTU6) or PKP3 knockdown clones (S9 and S10) were stained with phospho-specific antibodies to PRL3 (S73 and S431) and imaged by confocal microscopy. Note that the intensity of the signal for pS73 increases due to an increase in K8 levels in the PKP3 knockdown clones while the intensity of the signal for pS431 remains the same in the PKP3 knockdown clones. (Original magnification x 630 with 2X optical zoom. Scale bar 5 µm). B. The interaction between K8 and K18 is not altered upon PKP3 knockdown. The vector control (pTU6) or PKP3 knockdown clones (S9 and S10) were transfected with YFP-K18 and CFP-K8. 48 hours post transfection, FRET experiments were performed as described. The region in the white rectangle was bleached in the YFP channel and a corresponding increase was observed in the CFP channel as expected. FRET efficiencies were calculated for 5 different regions in three cells and results plotted in a bar graph. The FRET efficiency for the K18 K8 pair is comparable between the vector control and the PKP3 knockdown clones as shown in the graph. (TIF) [file pone.0038561.s004.tif]

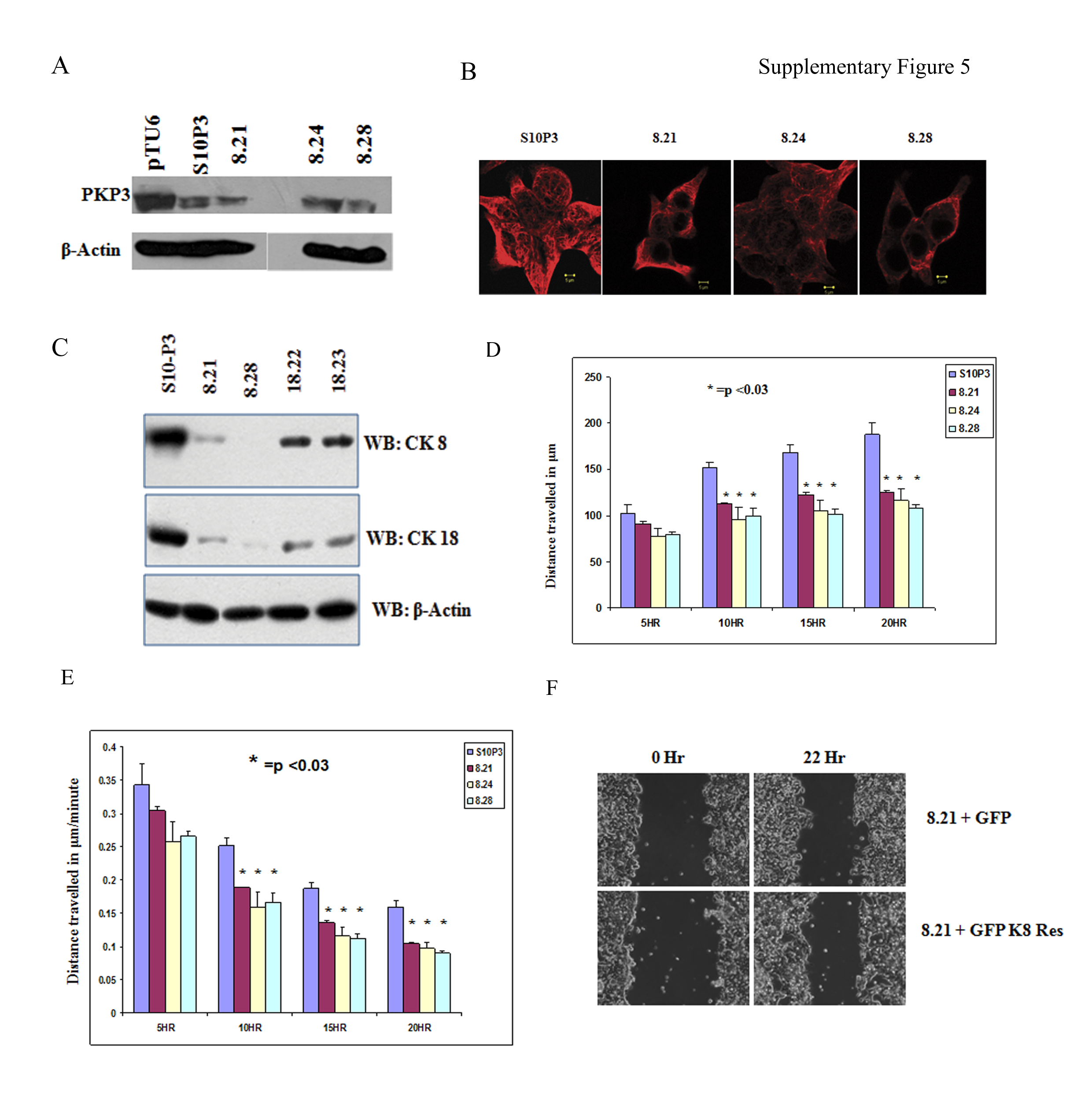

Supplement: Figure S5 — Characterization of K8 and PKP3 double knockdown clones. A. Generation of PKP3 and K8 double knockdown clones. Protein extracts from the S10 derived K8 (8.21, 8.24 and 8.28) knockdown clones or the vector alone (S10P3) or the vector control (pTU6) were resolved on SDSPAGE gels followed by Western blotting with antibodies to PKP3 and β-actin. B. The PKP3 K8 double knockdown clones or the PKP3 knockdown clone, were stained with antibodies against K8 (Original magnification x 630 with 2X optical zoom. Scale bars 5 µm). C. Protein extracts from the S10 derived K8 (8.21 and 8.28) or K18 knockdown clones (18.22 and 18.23) or the vector alone (S10P3) were resolved on SDSPAGE gels followed by Western blotting with antibodies to K8, K18, and β-actin. Note that K18 levels are low in the K8 knockdown clones while K8 levels are not altered substantially in the K18 knockdown clones. D. Scratch wound healing assays were performed on the double knockdown clones (8.21, 8.24 and 8.28) or the vector alone (S10P3). At different time points the distance migrated was measured and plotted as shown. The bars represent the mean of three independent experiments and the error bars represent the standard deviation. Statistical analysis was performed using two tailed students t test. Note that the distance migrated is significantly lowered in the double knockdown clones at 10, 15 and 20 hour time points. E. Scratch wound healing assays were performed on the double knockdown clones (8.21, 8.24 and 8.28) or the vector alone (S10P3). At different time points the distance migrated per minute was measured and plotted as shown. The bars represent the mean of three independent experiments and the error bars represent the standard deviation. Statistical analysis was performed using two tailed students t test. Note that the distance migrated is significantly lowered in the double knockdown clones at 10, 15 and 20 hour time points. F. Scratch wound healing assays were performed on the double knockdown c [file pone.0038561.s005.tif]

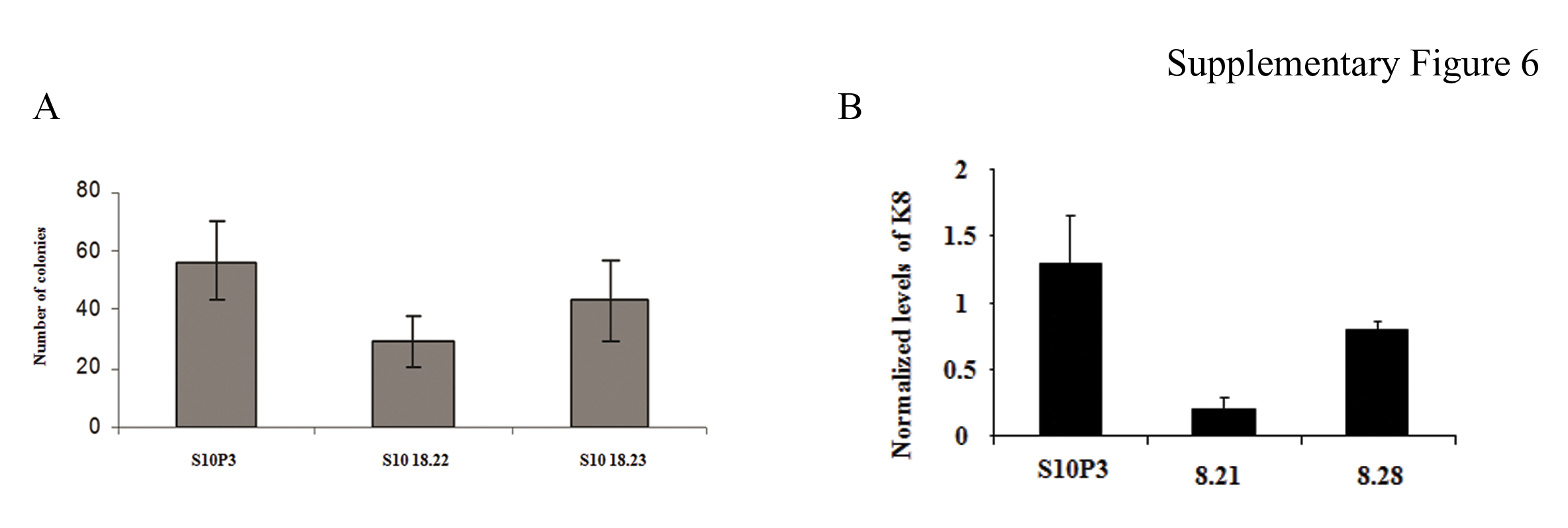

Supplement: Figure S6 — Transformation induced upon keratin loss in the PKP3 knockdown clones. A. The S10 derived K18 (18.22 and 18.23) knockdown clones or the vector alone (S10P3) were plated in soft agar and colony formation determined after 2–3 weeks. The number of colonies formed by the clones per 20 low power fields (10X) was counted in triplicate in each experiment and the mean and standard deviation of three independent experiments is plotted as shown. B. Densitometric analysis of K8 in tumor samples. Protein extracts derived from tumors generated upon injection of either the vector control (S10P3) or the double knockdown clones 8.21 and 8.28) were resolved on SDS PAGE gels followed by Western blotting Expression of K8 was normalized to that of β-actin and densitometry performed on Western blots using ImageJ software. Note that K8 levels are lower in the double knockdown clones when compared to the vector control. (TIF) [file pone.0038561.s006.tif]
